# Physical Activity Among Children With Congenital Heart Defects in Germany: A Nationwide Survey

**DOI:** 10.3389/fped.2020.00170

**Published:** 2020-04-30

**Authors:** Jannos Siaplaouras, Claudia Niessner, Paul C. Helm, Annika Jahn, Markus Flemming, Michael S. Urschitz, Elisabeth Sticker, Hashim Abdul-Khaliq, Ulrike M. Bauer, Christian Apitz

**Affiliations:** ^1^Division of Pediatric Cardiology, Children's Hospital, University of Ulm, Ulm, Germany; ^2^Institute of Sports and Sports Science, Institute of Technology, Karlsruhe, Germany; ^3^National Register for Congenital Heart Defects, DZHK (German Centre for Cardiovascular Research), Berlin, Germany; ^4^Coaching Competence Cooperation, Berlin, Germany; ^5^Abteilung für Pädiatrische Epidemiologie, Institut für Medizinische Biometrie, Epidemiologie und Informatik, Universitätsmedizin der Johannes Gutenberg-Universität Mainz, Mainz, Germany; ^6^Department Psychology, Faculty of Human Sciences, University of Cologne, Cologne, Germany; ^7^Klinik für Pädiatrische Kardiologie, Universitätsklinikum des Saarlandes, Homburg, Germany

**Keywords:** congenital heart defect (CHD), physical activities and sports, survey, pediatric cardiology, exercise limitation

## Abstract

**Objective:** In children with congenital heart defects (CHD), a sedentary lifestyle should be avoided and usually WHO recommendations on physical activity (PA) are supposed to be followed. In order to obtain representative data of the actual amount of PA (and potential influencing factors) in children with CHD we performed a nationwide online survey.

**Methods:** All patients aged 6–17 years registered in the German National Register for CHD were contacted by email and asked to participate in the survey using the comprehensive questionnaire of the “Motorik-Modul” from the German Health Interview and Examination Survey for Children and Adolescents (KiGGS), thus allowing the comparison with a representative age-matched subset of 3.385 participants of the KiGGS study. The questionnaire for CHD-patients was amended by specific questions regarding medical care, sports recommendations and PA restrictions.

**Results:** Complete datasets of 1.198 patients (mean age of 11.6 ± 3.1 years) were available for evaluation. Compared to the reference group, CHD patients significantly less frequently reached the WHO recommended level of 60 min of daily PA (8.8 vs. 12%; *p* < 0.001). Enjoyment in sports was almost equally distributed across CHD and reference groups, and strongly correlated with the level of PA (*r* = 0.41; *p* < 0.001). Remarkably, 49.2% of children with complex CHD, 31.7% with moderate, and even 13.1% with simple CHD were advised by their physician to restrict PA.

**Conclusions:** According to this nationwide survey, PA is markedly reduced in children with CHD. An important reason for this might be an unexpected high rate of physician-recommended restrictions on levels of PA.

## Introduction

Occurring at a rate of 1.1% out of all newborns, congenital heart defects (CHD) are the most frequent congenital malformation diagnosed in children (1). Due to recent improvements in surgical and interventional techniques as well as perioperative intensive care management, survival of children with CHD has markedly improved during the last decades, resulting in a growing number to survive to adulthood (2). As CHD patients get older, their cardiac health can additionally be affected by acquired cardiovascular risk factors (i.e., arterial hypertension, obesity, diabetes) commonly seen in the general population, thus increasing the risk of metabolic disease, stroke and coronary artery disease (3, 4). In fact, a recent study has suggested that myocardial infarction will become the leading cause of death in CHD patients with simple cardiac defects (5). It is known, that development of arteriosclerotic and metabolic disease manifesting in adulthood usually starts already in early childhood. Childhood obesity and sedentary lifestyle are known to represent major contributing factors (5). This highlights the need for primary prevention, hence lifestyle interventions are required to promote physical activity (PA) of pediatric CHD-patients (6, 7). Not to mention, that PA is indispensable for physical, emotional, and psychosocial development of children (8–10).

Thus, there is emerging consensus that children and adolescents with CHD should be encouraged to adopt a physically active lifestyle, and consequently, current sports recommendations for the majority of patients with simple and moderate CHD include participation in competitive sports, leisure sports and PA unrestricted following the World Health Organisation (WHO) recommendations for healthy children, i.e., daily participation of 60 min in moderate-to-vigorous PA that is developmentally appropriate and enjoyable (11). Complex CHD often requires more specific recommendations yet still with the aim to enable a physically active lifestyle.

Whether these recommendations on PA in children with CHD are adequately considered and generally implemented remained unanswered to this day. Previous studies investigating PA in children with CHD showed rather conflicting results. While some authors reported of reduced PA, especially in complex CHD, others revealed similar PA-levels compared to healthy controls (12–20). Discrepancies of results might be explained by differences in study designs and PA assessment tools, and furthermore, common to all of these studies are relatively small patient numbers included.

Therefore, we conducted a nationwide survey in collaboration with the German National Register for CHD (NRCHD) in order (I) to obtain representative data regarding the real world situation of the amount of PA and sports participation and its impact on physical self-perception in children with CHD living in Germany, (II) to detect differences compared to children without CHD using an appropriate reference cohort, and (III) to study factors potentially influencing PA and sports participation in CHD patients.

## Materials and Methods

### Study Design

This cross-sectional online survey was conducted from January to March 2018. Participants were recruited via the patient database of the NRCHD, the largest European registry for CHD patients (21). For patient recruitment, the database was searched for patients aged between 6 and 17 years at the time of the survey. Respective individuals and their parents were contacted and invited to take part in the survey via email. Ethical approval was obtained by the institutional ethical committee. The study protocol conforms to the ethical guidelines of the 1975 Declaration of Helsinki.

### Survey Instruments

PA and sports participation were assessed using the validated comprehensive questionnaire of the “Motorik-Modul” (MoMo) from the German Health Interview and Examination Survey for Children and Adolescents (KiGGS), thus allowing the comparison of obtained data with a representative age-matched subset of 3.385 same-aged participants of the MoMo wave 2 study (2015–2017) (22). Design and results of the MoMo Baseline and Longitudinal Study and details on the structure and content of the MoMo Physical Activity Questionnaire (MoMo-PAQ) have been published previously (22–25). Briefly, it consists of 28 items regarding frequency, duration and intensity of PA to capture habitual PA in different domains (PA in sports clubs, leisure time PA outside of sports clubs, extra-curricular PA, outdoor play, active commuting to school). Furthermore, the MoMo-PAQ consists of 36 items, which assess physical self-description based on the German version of the Physical Self-Description Questionnaire with answer categories to a 4-point Likert Scale (26). These 36 items represent the basic functions of physical performance: strength, endurance, speed, skills, coordination, and flexibility.

In addition, MoMo-PAQ included the German version of the Physical Activity Enjoyment Scale for children and adolescents (PACES) (27). The scale consists of 16 items (nine positive poled items, seven negative poled items) with answer categories to a 5-point Likert Scale.

The questionnaire for CHD-patients was amended by eight specific questions capturing the medical background of sports recommendations, sports restrictions and access to sports.

### Statistical Analysis

Values of continuous variables are reported as mean ± standard deviation. The Pearson's chi-square test was used for group comparisons including nominal data (e.g., gender and age). In order to assess the impact of potential contributing factors on PA, analysis of variance and analysis of covariance, Pearson's correlation as well as multiple and linear regression analysis was used, as appropriate. IBM SPSS statistics version 25.0 (IBM Inc., Armonk, NY, USA) was used for statistical analyses. A significance level of *p* ≤ 0.05 was applied.

## Results

### Patient Characteristics

Of 21.354 eligible patients, invitation was successfully delivered to 14.496 patients. 1.718 patients decided to participate in the study. Only complete datasets were considered for evaluation and were available from 1.198 CHD patients with a mean age of 11.6 ± 3.1 years. Of those 53.8% were male, and 46.2% female. The study participants were allocated into simple, moderate and complex CHD classification according to Warnes et al. (28) (Table 1). Thus, 411 (34.3%) were classified as simple CHD, 423 (35.3%) moderate, and 364 (30.4%) as complex CHD. Included patients had untreated CHD in 49.3%, 1–3 operations/interventions in 30.2%, and more than three operations/interventions in 20.5%. Genetic syndromes and chromosomal disorders were present in 70 patients (5.8%), most frequently trisomy 21 in 37 patients (3.1%) and Di-George-Syndrome in 15 patients (1.3%). Of all patients, 57.2% stated to live in an urban or suburban environment, whereas 42.8% live in rural areas. Patient characteristics of the study group did not significantly differ to the entire cohort of eligible patients.

**Table 1:** Classification of CHD severity according to Warnes et al. (28).

**Simple CHD**	**Moderate CHD**	**Complex CHD**
Isolated congenital aortic valve disease	Aorto–left ventricular fistulas	Conduits, valved, or nonvalved
Isolated congenital mitral valve disease (e.g., except parachute valve, cleft leaflet)	Anomalous pulmonary venous drainage, partial or total	Cyanotic congenital heart (all forms)
Small atrial septal defect	Atrioventricular septal defects (partial or complete)	Double-outlet ventricle
Isolated small ventricular septal defect (no associated lesions)	Coarctation of the aorta	Eisenmenger syndrome
Mild pulmonary stenosis	Ebstein's anomaly	Fontan procedure
Small patent ductus arteriosus	Infundibular right ventricular outflow obstruction of significance	Mitral atresia
Previously ligated or occluded ductus arteriosus	Ostium primum atrial septal defect	Single ventricle (also called double inlet or outlet, common, or primitive)
Repaired secundum or sinus venosus atrial septal defect without residua	Patent ductus arteriosus (not closed)	Pulmonary atresia (all forms)
Repaired ventricular septal defect without residua	Pulmonary valve regurgitation (moderate to severe)	Pulmonary vascular obstructive disease
	Pulmonary valve stenosis (moderate to severe)	Transposition of the great arteries
	Sinus of Valsalva fistula/aneurysm	Tricuspid atresia
	Sinus venosus atrial septal defect	Truncus arteriosus/hemitruncus
	Subvalvular AS or SupraAS (except HOCM)	Other abnormalities of atrioventricular or ventriculoarterial connection not included above (i.e., crisscross heart, isomerism, heterotaxy syndromes, ventricular inversion)
	Tetralogy of Fallot	
	Ventricular septal defect with: Absent valve or valves, Aortic regurgitation, Coarctation of the aorta, Mitral disease, Right ventricular outflow tract obstruction, Straddling tricuspid/mitral valve, Subaortic stenosis	

*CHDs were divided into simple, moderate and complex CHD according to the classification system by Warnes et al. (28). It is possible to be classified from a lighter group to a heavier group due to interventions and/or operations. Conversely, this is not possible. Therefore, this table shows only a rough schema of the severity classification. Taking all factors into account would be too extensive*.

### Physical and Sports Activity

**Figure 1:**
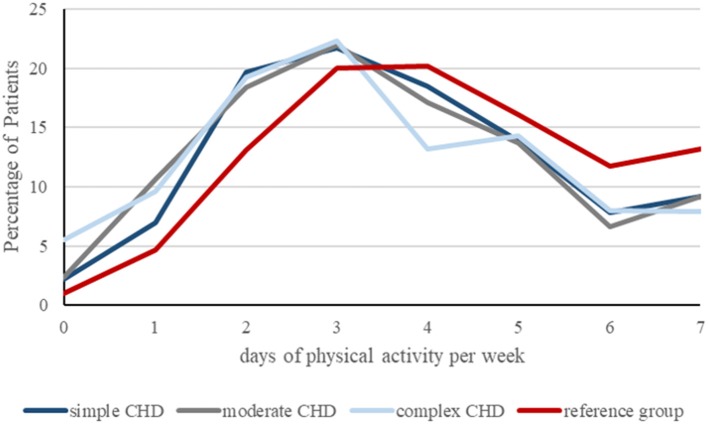
Graphical presentation of days of physical activity per week, achieved by patients with simple (*n* = 411), moderate (*n* = 423) and complex CHD (*n* = 364) compared to the reference group (*n* = 3.338) in percent. Children with CHD were 0.62 days per week less active than those of the reference group (*p* < 0.001).

Compared to MoMo participants, CHD patients reached significantly less frequently the WHO recommended level of 60 min of daily PA (8.8 vs. 12%; *p* < 0.001), simple CHD 9.2%, moderate 9.2%, and complex CHD 8.0%. Children with CHD were 0.62 days per week less active than those of the reference group (*p* < 0.001) (Figure 1).

Participation in sports clubs was significantly reduced in children with complex CHD compared to MoMo participants (53.6 vs. 67.6%; *p* < 0.001), whereas patients with simple and moderate CHD showed similar frequent participation as the reference group (74.9 and 66.2%, respectively). 61.4% of children with simple, 56.8% with moderate and 47.7% with complex CHD stated to participate even in competitive sports, compared to 53.1% of the reference group.

### Enjoyment in Sports

Enjoyment in sports was almost equally distributed across CHD groups and MoMo participants. Using the positive dimension of the PACES Scale ranging from 9 to 45 points we found 35.9 points for patients with simple CHD, 34.6 points for moderate and 33.2 points for patients with complex CHD, compared to 35.2 points for the reference group. Enjoyment in sports correlated with the level of PA (*r* = 0.41; *p* < 0.001).

### Physical Self-Description of Basic Functions of Physical Performance

**Figure 2:**
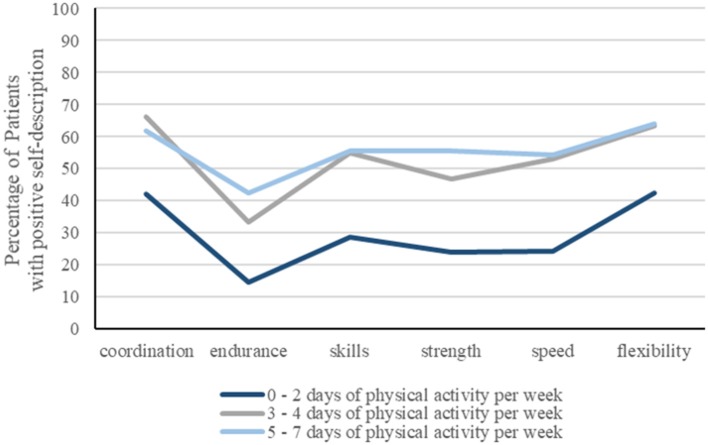
Relationship between positive physical self-description of basic functions of physical performance (strength, endurance, speed, skills, coordination, flexibility) with the level of physical activity (PA), expressed as days per week. Percentage of patients revealing a positive self-description is significantly higher when physically active on more than 2 days per week.

Physical self-description of all basic functions of physical performance (strength, endurance, speed, skills, coordination, flexibility) appeared to be related to the level of PA (Figure 2). More physically active patients showed higher percentage of positive self-description of basic functions of physical performance, most consistent for the dimension “endurance.”

**Figure 3:**
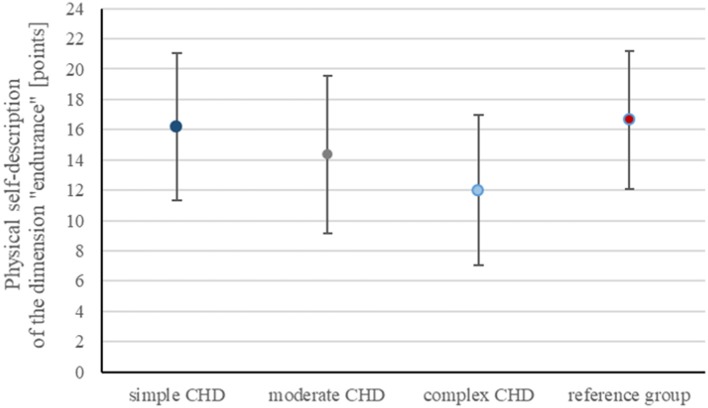
Graphical presentation of physical self-description of the dimension “endurance” (ranging from 6 to 24 points) provided as mean ± standard deviation showing reduction in CHD patients compared to the control cohort, most noticeable in complex CHD (11.9 ± 4.9 points) compared to 16.6 ± 4.5 points in the reference group.

Further analyzing the physical self-description of the dimension “endurance” (ranging from 6 to 24 points) showed significant reduction in CHD patients compared to the reference cohort (*p* < 0.001), most noticeable in complex CHD (11.9 ± 4.9 points) compared to 16.6 ± 4.5 points in the reference group (Figure 3). The dimension “endurance” correlated with the level of PA in all CHD groups (*p* < 0.001).

### Physician-Recommended Sports Restrictions

**Figure 4:**
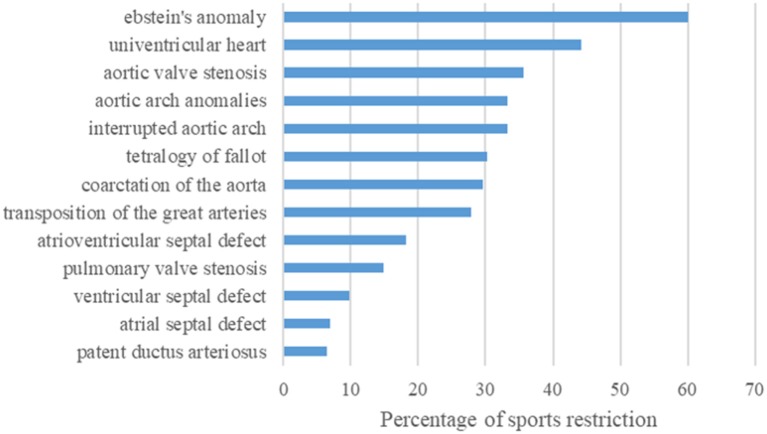
Bar graph showing the frequency of physician-recommended sports restriction in percent dependent on the corresponding CHD diagnosis. Of the 271 of 1.198 children with CHD receiving a sports restriction, most frequent restrictions have been advised in patients with Ebstein's anomaly, followed by single ventricle hemodynamics, and aortic valve stenosis.

49.2% of the children with complex CHD, 31.7% with moderate and even 13.1% with simple CHD were advised by their physician to restrict PA. 2.3% of all CHD patients received even a complete dispensation. Most frequently, sports restrictions have been advised in patients with Ebstein's anomaly, followed by single ventricle hemodynamics, and aortic valve stenosis (Figure 4). Interestingly, also patients with more simple CHD (i.e., ventricular septal defect or atrial septal defect) received sports restrictions. Advisory physicians in terms of sports were predominantly pediatric cardiologists (55.4%), followed by pediatricians (24.9%), family physicians (9.2%), orthopedists (2%), and sports medicine specialists (1.1%). In 7.4% the participant was not aware of the exact professional title of the advisory physician.

### Factors Influencing the Amount of Physical Activity

Regression analysis demonstrated a significant impact of age and gender on PA, whereas girls with CHD demonstrated lower levels of PA. Both, number of interventions and enjoyment in sports play a relevant role on the level of sports activity. Multiple regression analysis revealed physician-recommended PA restrictions having significant impact on the amount of PA, followed by sports activity of the father, whereas sports activity of the mother and residence in rural areas obviously had no significant impact on patient's PA (Table 2).

**Table 2:** Potential factors influencing physical and sports activity.

**Variable**	**Regression coefficient**	**Significance *p***
Age	−0.077^a^	<0.001
Gender	0.210^d^	0.050
Number of interventions	−0.056^b^	0.005
Residence in rural areas	−0.011	0.707
Sports activity father	0.216	0.047
Sports activity mother	0.017	0.571
Enjoyment in sports	0.103^c^	<0.001
Recommended restrictions	−0.294	0.012

a *Reduction of physical activity per year*.

b *Reduction of physical activity per intervention*.

c *Increase of physical activity per reached point in the PACES scale*.

d *Increase of physical activity of male children*.

## Discussion

To the best of our knowledge, the present study is the largest cohort of children with CHD that has been investigated for their physical and sports activity behavior. According to this nationwide survey, PA is markedly reduced in children with CHD living in Germany, and as expected, children with complex CHD showed the most relevant PA reduction. In addition, this study provides information on possible contributing factors that may influence the amount of PA beyond the burden of the heart defect itself, including role model function (regarding sports activity) of the father and an unexpected high rate of physician-recommended restrictions on PA levels.

Misjudgment in regard of risks vs. benefits of sports participation, as well as overprotection of parents or caregivers, as well as teachers and sport trainers have been previously reported as potential contributing factors (11, 29, 30). Our results suggest, that also physicians and health care professionals might tend to overprotect, when treating children with CHD, and this obviously often neither in accordance to current recommendations nor supported by scientific evidence, as it has previously been demonstrated that sudden death during exercise is extremely rare in CHD patients (31). Misperception is most notably when considering the relatively high rate of PA restrictions in simple CHD, i.e., patients who usually were allowed to perform unrestrictive leisure and competitive sports.

Recommendations for most patients with CHD include participation in competitive sport, leisure sport and PA unrestricted like healthy children, allowing participation in sports clubs and thus integration in normal social life, except for children with complex CHD or other risk factors (for example pacemaker, cardioverter-defibrillator, channelopathies) who frequently need specific individualized solutions. The latter can be covered by preventive cardiac rehabilitation groups organized by local pediatric heart centers or alternatively by individualized training programs. However, in Germany there are currently only eleven active regional cardiac rehabilitation groups for children with CHD compared to ~6,000 active cardiac rehabilitation groups for adults (32). Undoubtedly, this availability appears inadequate and is suggested to be influenced by differences in age, gender, interests, severity of CHD and exercise capacity, and additional logistical issues due to living in rural areas with long distances to the regional pediatric heart center. Indeed, our results were able to demonstrate that age, gender, enjoyment in sports and severity of CHD had significant impact on PA levels, however living in rural areas appears not to be a limiting factor, at least in a relatively small country as Germany.

Remarkably, our results show that children with CHD, even with complex CHD had well preserved enjoyment in sports on a similar level as the control group. This has to be considered and implicates that in children the motivation regarding PA and sports appears to be high, underlining the social implications of sports participation in childhood and supports the feasibility of individual exercise training in the pediatric age group. To maintain or even increase motivation, training programs designed for children should usually prefer varied activity tasks focusing mainly on skills, coordination, and speed, and avoid monotonous endurance training, as frequently used in adults (11, 33). The fact that reduced PA correlated with impaired physical self-perception demonstrates the negative psychological consequences of a sedentary lifestyle. This is alarming and might result in a downwards spiral of inactivity. Physicians should therefore regularly advice on patients' PA level and sports activity in every clinical consultation.

### Study Limitations

Data of this survey are based on self- or proxy-reports and might therefore be prone to bias, including recall bias and social desirability, as children with CHD may overestimate their PA level (34). Furthermore, the absence of direct contact with patients may leave the chance for misunderstanding of questions or incorrect answers. To prevent such occurrences each study participant was offered to contact the study management if having any questions. In addition, at the end of the survey there was the possibility to provide ambiguities and questions directly as free text. Clearly, measuring PA by objective methods (i.e., accelerometry, pedometers, activity trackers) might be more precise (14). However, the objective assessment of PA in different domains can be challenging and requires complex strategies, which can hardly be applied in large patient populations. The strength of the current study is the consideration of different domains of PA, the large sample size and the inclusion of school-aged children with a wide age range, as well as the representative reference group.

Since this is a cross-sectional study, we provide descriptive information and report on associations rather than claiming to report causal relationships between PA reduction and potential contributing factors (2). Moreover, the results reflect respondents' subjective statements. The results may not be applicable to CHD patients outside Germany, since they are affected by the life situation of the patients, as well as the organization of the health care and educational system (2).

Since the patients have been invited by emails to participate in the survey, the response rate of 11.9% was relatively low. Nevertheless, the sample of patients participating in the online survey did not differ significantly to the entire CHD cohort of NRCHD and therefore seems to be representative for the German CHD community at large.

Theoretically, by considering the prevalence of CHD in the German population of 1.1%, it cannot be ruled out that a minority of participants in the KiGGS study may also be affected by CHD. Due to inaccurate recording of CHD diagnoses in the KiGGS dataset a removal of these participants was not feasible. However, from a statistical point of view, having these children included in the reference group should not result in a relevant bias.

## Conclusions

According to this nationwide survey, PA is markedly reduced in children with CHD, at least in part due to an unexpected high number of physician-recommended PA restrictions. As sedentary lifestyle may have negative implications on cardiovascular risk profile and prognosis, future efforts should be directed toward facilitating the access to PA for all CHD patients.

## Data Availability Statement

All datasets generated for this study are included in the article/supplementary material.

## Ethics Statement

The studies involving human participants were reviewed and approved by Ethical Review board of the Charité, Berlin (Approval number 2/034/17). Written informed consent to participate in this study was provided by the participants' legal guardian/next of kin.

## Author Contributions

JS, CN, and CA conceptualized and designed the study, drafted the initial manuscript, and reviewed and revised the manuscript. UB, HA-K, ES, MU, MF, and AJ designed the data collection instruments, collected data, carried out the initial analyses, and reviewed and revised the manuscript. PH conceptualized and designed the study, coordinated and supervised data collection, and critically reviewed the manuscript for important intellectual content. All authors approved the final manuscript as submitted and agree to be accountable for all aspects of the work.

## Conflict of Interest

The authors declare that the research was conducted in the absence of any commercial or financial relationships that could be construed as a potential conflict of interest.

## Abbreviations

CHD: congenital heart defects
KiGGS: German Health Interview and Examination Survey for Children and Adolescents
MoMo: Motorik-Modul
NRCHD: National Register for congenital heart defects
PA: Physical Activity
PACES: Physical Activity Enjoyment Scale for children and adolescents
PAQ: Physical Activity Questionnaire
WHO: World Health Organization.
